# A Case of Lynch Syndrome-Associated Colorectal Adenocarcinoma in a 19-Year-Old Female Patient

**DOI:** 10.7759/cureus.48740

**Published:** 2023-11-13

**Authors:** Aimen Iqbal, Sandra K Rabat, Ravleen Kaur, Muhammad Waqas, Sanya Badar, Faryal Haider, Muneebuddin Syed, Linda Thomas

**Affiliations:** 1 Internal Medicine, The Wright Center for Graduate Medical Education, Scranton, USA; 2 Internal Medicine, Bhaskar Medical College, Hyderabad, IND

**Keywords:** young-onset colorectal cancer, mismatch repair, cancer immunotherapy, lynch syndrome, colorectal cancer

## Abstract

This case report presents the diagnostic journey and management of a 19-year-old female who was diagnosed with advanced colorectal cancer (CRC) associated with Lynch syndrome (LS), a hereditary nonpolyposis colorectal cancer (HNPCC). The patient initially presented with complaints of persistent abdominal pain, vomiting, and unexplained weight loss, leading to investigations revealing hypochromic microcytic anemia and the presence of an inhomogeneous pelvic mass associated with the sigmoid colon. Subsequent diagnostic procedures, including flexible sigmoidoscopy and pathology reports, confirmed the presence of an advanced rectosigmoid adenocarcinoma with high-grade dysplasia. Molecular testing and immunohistochemical staining revealed a deficiency in mismatch repair proteins, confirming the diagnosis of LS. Despite ineligibility for certain clinical trials due to lymph node infiltration, the patient demonstrated a significant positive response to pembrolizumab immunotherapy, with a notable reduction in tumor size and lymph node involvement. This case underscores the significance of genetic predisposition in the development of early-onset CRC and the potential efficacy of immunotherapy in managing advanced CRC associated with LS in young patients. Additionally, this case provides insights into the evolving landscape of CRC management and the critical role of personalized treatment strategies in optimizing patient outcomes.

## Introduction

Colorectal cancer (CRC) is the fourth most prevalent form of cancer in the United States, typically diagnosed at a median age of 68 years. Among the myriad factors contributing to its incidence, Lynch syndrome (LS), also known as hereditary nonpolyposis colorectal cancer (HNPCC), has emerged as a critical contributor, accounting for approximately 3% of all newly identified cases [1]. LS arises from germline mutations of the mismatch repair (MMR) genes, propelling an increased predisposition toward CRC development [2]. Of notable concern is the escalating prevalence of CRC within the adolescent and young adult (AYA) population, particularly those aged 18-40 years, as independently analyzed by prominent cancer databases such as the Surveillance, Epidemiology, and End Results (SEER) Program and the National Cancer Database (NCDB) in the United States [3-9]. Understanding the prominence and implications of LS becomes paramount in the context of this escalating trend. The manifestation of CRC occurs at a significantly younger age among patients afflicted with LS compared to those experiencing sporadic CRC, with age ranges typically falling between 45 and 60 years for LS and 69 years for sporadic cases. This divergence in age of onset accentuates the urgency of identifying and comprehensively addressing hereditary syndromes associated with CRC, with LS at the forefront, particularly in younger populations [10]. In this case report, we highlight a 19-year-old female patient diagnosed with stage IV CRC, stemming from underlying LS.

## Case presentation

A 19-year-old female with no significant past medical history presented to our outpatient clinic with a chief complaint of abdominal pain, vomiting, and diarrhea for the past four months. Upon her prior visits to the clinic, she reported similar symptoms; however, they were initially attributed to her known egg allergy, and investigations for malabsorption yielded negative results. She was noted to have unintentional weight loss that had started about a year ago, resulting in a total weight reduction of approximately 25 pounds. Furthermore, she reported a decreased appetite and intermittent episodes of nausea and vomiting. Initially, she had been diagnosed with a urinary tract infection and demonstrated improvement in her symptoms following the completion of a prescribed antibiotic course; however, her symptoms did not completely resolve. Concurrently, she also began to experience more frequent and, at times, explosive diarrhea but denied hematochezia and melena. 

She underwent further workup with laboratory and diagnostic studies. Laboratory results demonstrated hypochromic microcytic anemia, particularly iron deficiency anemia (Table 1).

**Table 1:** Laboratory results

Test	Result	Reference range	Unit
Hemoglobin	9.6	12.0-15.0	g/dL
Hematocrit	33.6	36.0-45.0	%
Mean corpuscular volume	73	80-100	fL
Mean corpuscular hemoglobin	21.8	26-33	pg
Mean corpuscular hemoglobin concentration	29.7	32-36	g/dL
Platelets	518	140-400	K/uL
Iron	12	50-212	µg/dL
Transferrin	175	203-362	mg/dL
Ferritin	40.0	11.0-306.8	ng/mL
Transferrin saturation	5	20-50	%
Total iron binding capacity	245	260-430	µg/dL

**Figure 1:**
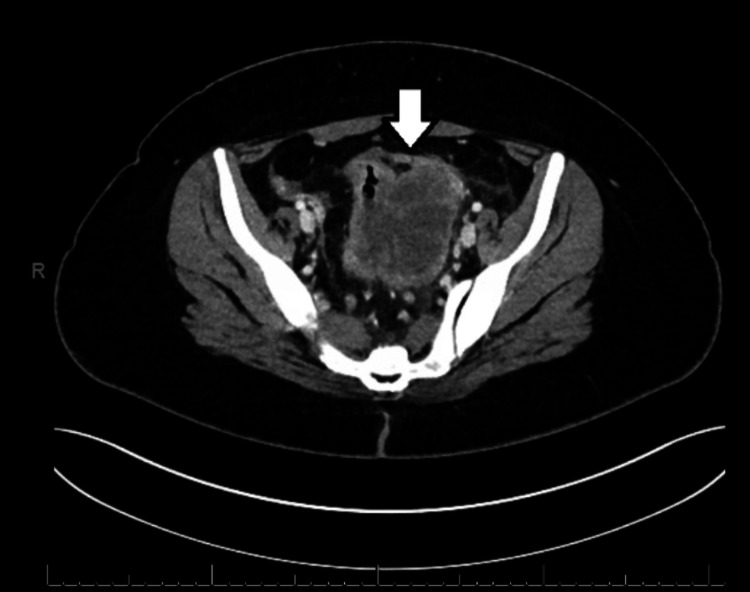
CT scan of the abdomen/pelvis demonstrating an inhomogeneous mass within the mid to left pelvis in transverse view

**Figure 2:**
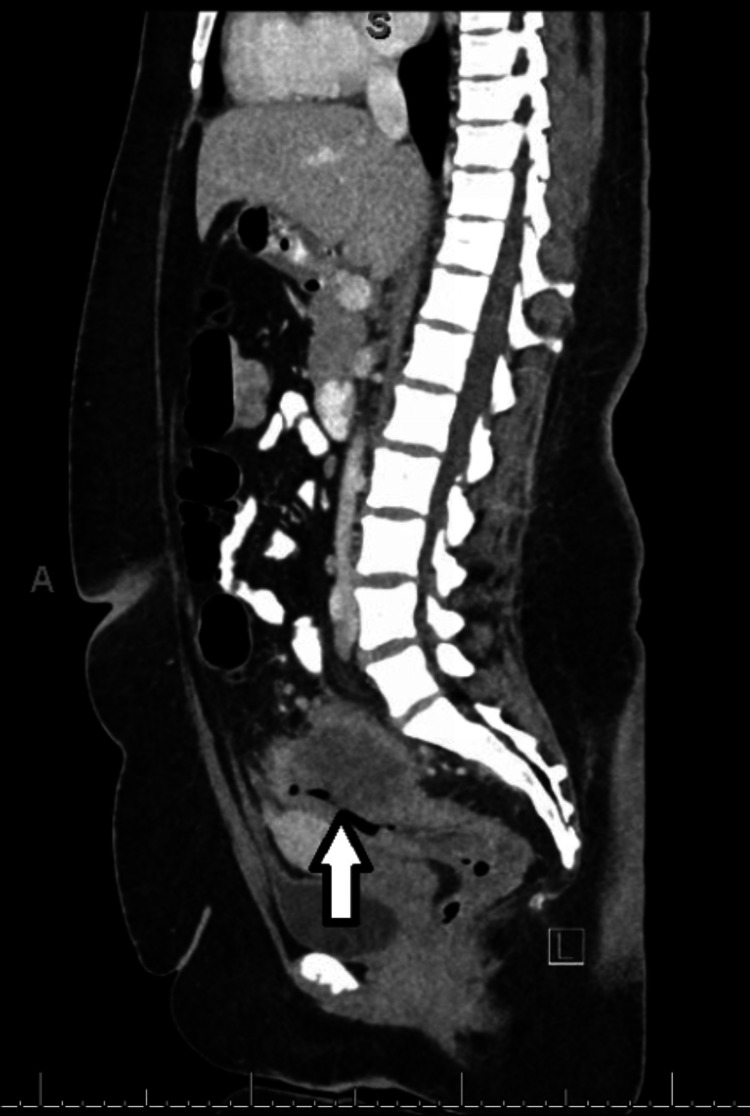
CT scan of the abdomen/pelvis demonstrating an inhomogeneous mass within the mid to left pelvis in vertical view

An initial abdominal ultrasound was performed, revealing the presence of cholelithiasis, albeit without notable gallbladder wall thickening, pericholecystic fluid, or sonographic Murphy sign. Furthermore, the examination noted a mild enlargement of the liver, with limited visibility of the pancreas and abdominal aorta due to the presence of overlying bowel gas. Subsequently, the patient was assessed for a colonoscopy, but the procedure was prematurely terminated due to the presence of bowel stenosis. During the procedure, diffuse and severe mucosal alterations were identified in the rectosigmoid colon. A large polyp was also discovered in the rectosigmoid colon. Biopsies were obtained from the affected areas. The pathology of the rectosigmoid biopsy confirmed at least high-grade dysplasia/intramucosal carcinoma associated with tubulovillous adenoma with high-grade dysplasia. There was a note that a more advanced lesion cannot be excluded due to superficial sampling. A computed tomography (CT) scan of the abdomen/pelvis revealed an approximate 6.4 × 7.7 × 6.7 cm inhomogeneous mass within the mid to left pelvis closely associated with the left superolateral aspect of the mid-sigmoid colon (but also closely applied to the left ovary), as shown in Figures 1, 2. The sigmoid colon was under-distended with wall thickening, with a few small mesenteric and retroperitoneal lymph nodes identified and a few small lymph nodes in the presacral fat.

**Figure 3:**
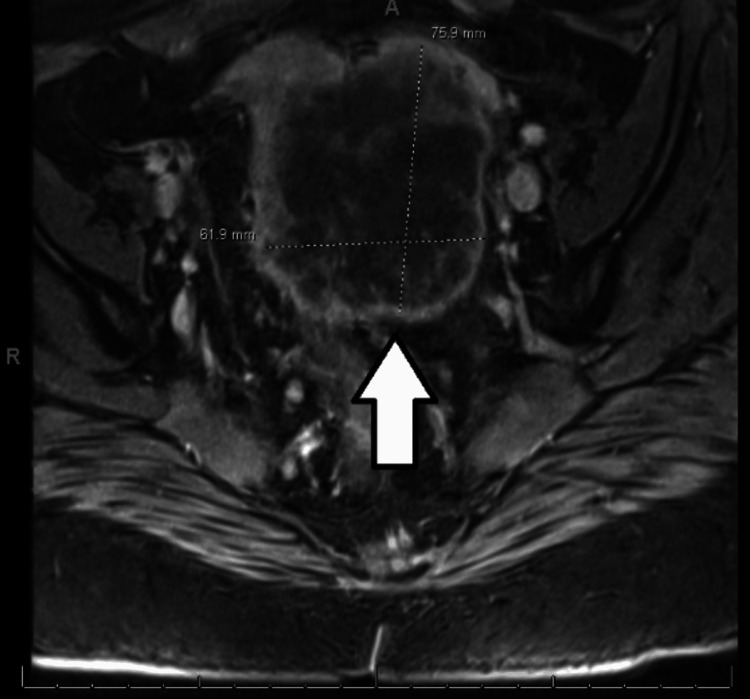
MRI of the pelvis without contrast demonstrating a large mass measuring 7.6 × 6.2 × 6.2 cm present in the pelvis predominantly to the left of the midline

She had a magnetic resonance imaging (MRI) of the pelvis without contrast that showed a large mass measuring 7.6 × 6.2 × 6.2 cm present in the pelvis predominantly to the left of the midline, which was invading the sigmoid colon and extending into the mesorectal fascia to the right of the midline (Figure 3). The mass demonstrated heterogeneous enhancement with peripheral predominant vascularity. The inferior-most edge of the mass was 16 cm from the anal verge. The mass invaded the left broad ligament and the left adnexa with left lateral displacement of the left ovary. There was no gross invasion of the right ovary. Multiple mesorectal lymph nodes were present with the largest measuring 9 mm. Multiple retroperitoneal lymph nodes were also present with the largest measuring 9 mm just anterior to the bifurcation of the aorta.

The patient was evaluated by general surgery and subsequently underwent a flexible sigmoidoscopy, which identified a partially obstructing mass involving the proximal rectum. The biopsy pathology report revealed intramucosal adenocarcinoma in a superficial fragment of a tubulovillous adenoma. There was a note that due to the superficial fragmented nature of the biopsy, the exact depth of invasion could not be definitively ascertained. Carcinoembryonic antigen (CEA) levels were 17 ng/mL (≤5 ng/mL). Positron emission tomography/CT (PET/CT) revealed fluorodeoxyglucose (FDG) avid lobulated exophytic rectosigmoid mass with FDG avid mid to distal rectal thickening, consistent with primary neoplasm, FDG avid retroperitoneal and pelvic nodes consistent with metastatic disease, and focal right pelvic peritoneal nodularity uptake, indeterminate. Additional findings include diffusely FDG avid spleen and axial/proximal appendicular marrow, probably reactive, and a few subcentimeter short-axis cervical nodes with low-grade FDG uptake, possibly reactive. PET scan classified the tumor as T4bN1M1 disease. 

For HNPCC screening, immunohistochemical (IHC) staining was done. There was a loss of nuclear expression of MutL homolog 1 (MLH1) and post-meiotic segregation increased 2 (PMS2). Next-generation sequencing (NGS) revealed the following results, as listed in Table 2.

**Table 2:** Next-generation sequencing

Gene	Variant	Consequence
KRAS	A59T	Missense variant
KRAS	G12D	Missense variant
APC	H298Lfs*28	Frameshift variant
ARID1A	Q1452Rfs*29	Frameshift variant
ERBB3	V104M	Missense variant
PIK3R1	K567E	Missense variant

Additionally, the microsatellite instability (MSI) status was high.

The patient reported no family history of cancer in her parents and siblings but reported a history of esophageal cancer in her maternal cousin, who had a history of alcohol and substance use disorder. The patient and her family chose to pursue treatment at an external medical facility. Unfortunately, the patient did not qualify for participation in clinical trials at that particular facility due to the tumor’s infiltration into neighboring lymph nodes. However, she remained eligible to receive immunotherapy with pembrolizumab (anti-PD1). In preparation for systemic immunotherapy administration, the patient underwent a port-a-cath placement. Approximately four months later, the patient returned to our clinic for a follow-up appointment. During this period, she completed four sessions of immunotherapy. The patient reported a significant positive response to the treatment, noting a remarkable reduction in tumor size by 50% and a 25% reduction in lymph node involvement.

## Discussion

Genetics

LS presents a distinctive phenotype marked by a higher incidence of tumors, primarily on the right side of the colon, along with a notable inclination toward synchronous and metachronous CRCs [5,11]. Diagnosing LS involves the identification of specific genetic anomalies, such as a germline mutation in one of the mismatch repair genes, including MLH1, MSH2, MSH6, or PMS2, or a germline deletion in EpCAM (epithelial-cell adhesion molecule), which results in the epigenetic inactivation of MSH2 [11,12]. Our patient had a loss of nuclear expression of MLH1 and PMS2. When a mutation occurs in one of the mismatch repair genes within the germline, there exists a significant likelihood of a subsequent somatic mutation occurring in the other copy of that gene, compromising the mismatch repair function and ultimately paving the way for cancer development. This impaired mismatch repair mechanism subsequently leads to MSI within the affected tumors, evidenced by changes in the lengths of repetitive regions within the DNA, known as microsatellites [13].

Approximately 60-80% of all cancers associated with LS have been linked to mutations in MLH1 or MSH2, while the remaining cases are ascribed to MSH6 or PMS2, with EpCAM mutations being a rare occurrence [11,14]. Individuals with mutations in MSH2 exhibit a heightened susceptibility to extracolonic cancers, particularly endometrial cancer [15]. Recent population-based data have revealed a relatively higher prevalence of germline mutations in MSH6 and PMS2 than MLH1 or MSH2, although with a comparatively lower likelihood of cancer development, including CRC. It is worth noting that carriers of MSH6 mutations tend to manifest with CRC and endometrial cancer at more advanced ages as compared to carriers of MLH1 or MSH2 mutations [14,15].

It’s essential to recognize that deficient mismatch repair and the resulting MSI are not unique to LS alone. These anomalies are more commonly observed in sporadic cases of CRC triggered by MLH1 promoter hypermethylation or, less frequently, by double somatic mismatch repair mutations [16,17]. It has been observed that sporadic CRCs with deficient mismatch repair are more prevalent in women and are typically diagnosed in patients at a more advanced age compared to cancers associated with LS [16].

Young-onset CRC has been postulated to arise from a complex interplay between genetic alterations that confer elevated susceptibility and environmental influences, emphasizing the multifactorial nature of its etiology [18]. Despite the majority of CRC cases occurring sporadically, there is substantial evidence suggesting a strong genetic component contributing to its pathogenesis [19]. One of the extensively studied genetic subtypes of CRC involves the aberrant mismatch repair pathway, often accompanied by MSI, underscoring the intricate molecular mechanisms involved in the development of this malignancy [20].

The role of the K-RAS oncogene, a key player in the kinase signaling growth pathway, cannot be overlooked in the context of CRC pathogenesis [21]. NGS of our patient revealed two missense variants in the KRAS oncogene. New findings have shown that some children can get CRC early if they have certain changes in their MMR genes, showing how the disease can vary [22-24]. Goel et al. reported in 2010 that the somatic mutation of the K-RAS gene, particularly missense mutations at codons 12 and 13, has been comparatively less frequently observed in CRC cases associated with LS than in sporadic CRC, indicating potential distinctions in the underlying molecular pathways between the two cohorts [25]. While the precise effect of K-RAS gene mutations on the timing and prognosis of CRC remains uncertain [26], it is conceivable that the combined impact of the K-RAS mutation on the growth factor signaling pathway, in conjunction with the MMR gene mutation, may potentially act synergistically to promote the onset of CRC at an earlier age. The decreasing age at the diagnosis of CRC in successive generations of Lynch families suggests a potential evolving genetic predisposition within these lineages [27]. 

Clinicopathologic features

The landscape of CRC in the AYA population, referred to as young-onset CRC, presents distinct characteristics compared to its older counterpart. Notably, this form of CRC displays a predilection for the distal colon and rectum, as consistently indicated by the SEER and NCDB analyses [28,29]. In our patient, CRC involved the proximal rectum. 

Young-onset CRC tends to exhibit more advanced disease at the time of diagnosis, with a higher incidence of stage III or IV cancers compared to older patients, particularly in cases affecting the colon or rectum [30]. Several distinguishing features, including larger tumor size, increased rates of perineural or lymphovascular invasion, and specific histological types like signet cell or mucinous, further characterize this subset of CRC cases [29]. Our patient had an initial diagnosis of stage IV.

In the context of LS-related CRC, the disease manifests at a younger age, primarily affecting the right colon, highlighting the distinct clinical pattern associated with this hereditary condition [31]. Emerging evidence suggests a significant association between obesity, as indicated by a body mass index (BMI) of ≥30 kg/m2, and an increased risk of young-onset CRC, with higher BMIs during early adulthood and significant weight gain during adulthood further compounding this risk [32]. Our patient had a BMI of 35.4 kg/m2. 

Additionally, a subset of young-onset CRC cases is linked to underlying inflammatory bowel diseases such as Crohn’s colitis and ulcerative colitis, which confer varying degrees of CRC risk over the duration of the disease, with a higher risk observed in cases of pancolitis [33]. Symptomatically, the inclination toward the distal colon and rectum in young-onset CRC translates into characteristic clinical presentations, including rectal bleeding, rectal pain, altered bowel habits, bloating, abdominal pain, and episodes of nausea and vomiting [34,35]. Our patient demonstrated similar symptoms of altered bowel habits, abdominal pains, and episodes of nausea, which were initially attributed to her egg allergy. 

Diagnosis

The diagnosis and management of CRC in young adults present unique challenges. In many cases, young-onset CRC is detected after the manifestation of symptoms, leading to delays in both presentation and treatment initiation compared to older adults. This delay, often up to 6.2 months from symptom onset, is exacerbated by the frequent misattribution of symptoms to benign anorectal conditions like hemorrhoids, without the necessary follow-up investigations. Factors such as underinsurance and hesitancy to seek medical care among the AYA population contribute to late-stage presentations of young-onset CRC, emphasizing the critical need for a high level of suspicion and prompt diagnostic workup in this patient group [3,4,9,35,36-38].

Key components of the diagnostic workup include obtaining a histologic diagnosis through an endoscopic biopsy, with a full colonoscopy as the preferred method for assessing the entire colon. However, in cases of obstructing distal tumors, alternative approaches may be necessary. A comprehensive oncologic staging should follow, involving physical examinations, laboratory studies, and imaging studies for local assessment of rectal cancer. Concurrently, evaluating potential hereditary CRC syndromes is paramount, requiring a thorough examination for extracolonic manifestations and a detailed family cancer history [39-43]. Patients should be referred for genetic counseling and risk assessment, followed by appropriate germline testing of specific genes or a combination of genes as required [44].

Identifying LS, an autosomal dominant hereditary CRC syndrome, remains a challenge due to insufficient awareness and inadequate assessment of family history. Recognizing the importance of accurate identification, guidelines such as the Amsterdam criteria and the Bethesda guidelines have been developed to aid in the diagnosis, with recommendations for universal testing of all newly diagnosed CRC patients for deficient mismatch repair or MSI to ascertain the presence of the LS. IHC analysis and polymerase-chain-reaction-based testing play pivotal roles in this process, with the loss of mismatch repair proteins directing subsequent germline testing of the implicated genes [45-48].

In cases of CRC, the presence or absence of MSI serves as an indicator of the efficiency of DNA mismatch repair. The detection of specific markers like MLH1 and PMS2 expression, as well as the BRAF V600E mutation and MLH1 hypermethylation status, plays a crucial role in identifying the cancer’s origin. If the BRAF V600E mutation or MLH1 hypermethylation is present, it suggests a sporadic origin. Conversely, individuals displaying the loss of MSH2, MSH6, or PMS2, or high MSI, should consider cancer genetic counseling and undergo germline genetic testing to confirm the presence of LS [49]. The integration of NGS of tumor tissue as an initial diagnostic step has shown promise in simplifying the evaluation process for LS. Following the identification of a germline mutation in a mismatch repair gene, genetic testing for at-risk family members, particularly first-degree relatives, is strongly recommended, highlighting the significance of early detection and preventive measures within affected families [50-52]. 

Screening

Screening for CRC before the age of 50 is crucial for individuals deemed to be at elevated risk. The National Comprehensive Cancer Network (NCCN) guidelines emphasize earlier initiation of screening for patients with major hereditary CRC syndromes and inflammatory bowel disease, underscoring the importance of tailored approaches to high-risk populations [53]. Furthermore, a pertinent personal or family history of the disease necessitates vigilant monitoring. For instance, individuals with a family history of advanced adenoma or CRC in one first-degree relative before the age of 60, or in two first-degree relatives at any age, should consider commencing screening colonoscopy 10 years before the age of diagnosis of the youngest affected relative or at the age of 40, depending on which comes first [54,55]. In addition, individuals with the germline mutation in a mismatch repair gene have a 50% chance of passing it on to their children, thereby warranting comprehensive counseling and risk management strategies. For carriers of the mutation, regular colonoscopy every one to two years is strongly recommended, beginning as early as 20-25 years of age, or two to five years before the youngest age at which CRC was diagnosed in the family, especially if the diagnosis occurred before 25 years of age. Similarly, for carriers of MSH6 or PMS2, colonoscopy every one to two years should be initiated at the ages of 30 and 35, respectively, acknowledging the specific risks associated with different gene mutations [52].

The 2018 qualified recommendation by the American Cancer Society to initiate average-risk screening at the age of 45 signifies a proactive shift in screening guidelines, emphasizing the importance of early detection and intervention in mitigating the risk of CRC and improving patient outcomes [55]. 

Management

In the realm of both young-onset and later-onset stage IV CRC, the established standard of care primarily involves a first-line therapy regimen that integrates either an oxaliplatin- or irinotecan-based multiagent approach alongside a biologic agent [39]. Developments in ongoing clinical trials have brought to light the potential of immunotherapy in revolutionizing the management of metastatic stage IV CRC, particularly in the context of tumors exhibiting MSI. The use of antibodies targeting PD1, PDL1, and CTLA4 has demonstrated remarkable and dramatic responses, offering new avenues for the treatment of otherwise challenging and aggressive forms of CRC [40,56]. The guidelines outlined by the U.S. Multi-Society Task Force on Colorectal Cancer underscore the significance of colectomy with ileorectal anastomosis as the primary surgical approach for individuals diagnosed with LS and CRC. However, the consideration of less extensive surgical options for patients aged over 60-65 years and those with underlying sphincter dysfunction is crucial, taking into account the potential risks associated with chronic diarrhea and incontinence. Subsequent endoscopic surveillance of the remaining rectum at regular intervals, typically every six or 12 months post-subtotal colectomy, is recommended to ensure comprehensive monitoring and early detection of any potential disease recurrence, emphasizing the importance of tailored and individualized treatment strategies in optimizing long-term outcomes and patient well-being [52].

CRCs characterized by deficient mismatch repair exhibit a distinct immunogenic profile characterized by abundant frameshift mutation-specific neoantigens, triggering an increased density of tumor-infiltrating lymphocytes. Despite this heightened immunogenicity, the inability of T cells to effectively eradicate these tumors can be attributed, in part, to the overexpression of immune checkpoint proteins that can be targeted by checkpoint inhibitors. The evaluation of anti-programmed death 1 (PD-1) antibodies, including pembrolizumab and nivolumab, in patients with metastatic CRC and deficient mismatch repair, refractory to prior cytotoxic agents, has demonstrated promising objective response rates ranging from 31% to 52%, with sustained responses and prolonged median progression-free survival and overall survival rates yet to be reached. Encouragingly, response rates were consistent across patients with both LS-associated CRCs and non-LS-associated CRCs, underscoring the potential efficacy of these therapeutic agents in diverse patient populations [57-62].

The endorsement of universal testing for deficient mismatch repair or MSI in all newly diagnosed CRCs by leading oncology organizations, such as the American Society of Clinical Oncology, the American Society for Clinical Pathology, the Association for Molecular Pathology, the College of American Pathologists, the NCCN, and the European Society for Medical Oncology, reflects the growing recognition of the importance of comprehensive molecular profiling in informing tailored treatment strategies and optimizing patient outcomes. Additionally, the incorporation of pembrolizumab or nivolumab as second-line or later treatment options for metastatic CRCs with deficient mismatch repair, as recommended by NCCN guidelines, highlights the evolving treatment landscape and the potential of immunotherapy in reshaping the management of advanced CRC, thus underscoring the significance of integrating molecular testing and targeted therapies in contemporary oncology practice [39].

The psychosocial well-being of AYA patients with CRC remains a critical area of focus, with psychosocial support and engagement within AYA patient communities playing a pivotal role in addressing the unique challenges faced by this patient demographic. Given the potential limitations in life experiences and coping skills among AYA patients, the provision of comprehensive psychosocial support services is crucial in fostering a supportive environment and facilitating effective coping mechanisms throughout the treatment journey [63]. The long-term survivors of young-onset CRC encounter persistent challenges, including heightened levels of anxiety, body image concerns, and embarrassment associated with bowel movements, when compared to their older counterparts. The impact of CRC on various aspects of survivors’ lives, including role functioning, social interactions, emotional well-being, and cognitive abilities, underscores the complex and multifaceted nature of the psychosocial implications of CRC. Specific symptoms such as constipation, diarrhea, fatigue, and insomnia further contribute to the burden experienced by survivors, highlighting the necessity for tailored and holistic psychosocial support programs to address the diverse needs of this patient population [63,64].

In conjunction with psychosocial support, addressing the comprehensive needs of AYA patients with CRC involves a multidisciplinary approach encompassing oncofertility and family planning, financial and employment counseling, and holistic approaches to nutrition and integrative medicine. Proactive integration of these supportive services is essential in fostering a holistic and patient-centered approach to cancer care, thereby optimizing the overall well-being and quality of life of AYA patients and ensuring that their physical, emotional, and psychosocial needs are effectively addressed throughout the continuum of care.

## Conclusions

To sum up, this case report highlights how genetic factors, especially LS, can play a significant role in causing CRC, especially in young adults. It emphasizes the importance of recognizing genetic risks early and taking proactive steps for better management. It also underlines the need for better awareness and support for young patients dealing with CRC, addressing not just the medical aspects but also the emotional and social challenges they face. This case stresses the significance of personalized care and early intervention in managing hereditary conditions as well as the importance of tailored treatments for better outcomes, particularly in young adults.
